# The accuracy of pre-appendectomy computed tomography with histopathological correlation: a clinical audit, case discussion and evaluation of the literature

**DOI:** 10.1007/s10140-014-1243-z

**Published:** 2014-05-31

**Authors:** George Benjamin Collins, Tien Jin Tan, John Gifford, Andrew Tan

**Affiliations:** 1 Addenbrooke’s Hospital, Cambridge University Hospitals NHS Foundation Trust, Cambridge Biomedical Campus, Hills Road, Cambridge, CB2 0QQ UK; 2 Department of Radiology, Changi General Hospital, 2 Simei Street 3, Singapore, 529889 Singapore

**Keywords:** Acute appendicitis, Computed tomography, Acute abdomen, Abdominal pain, Appendix, Clinical audit

## Abstract

The increasing use of computed tomography (CT) in acute appendicitis makes recognising the radiological hallmarks of the condition and its mimics vital. The differential diagnosis includes both appendiceal and nonappendiceal pathologies. The correlation between pre-appendectomy CT and post-appendectomy histopathology was audited retrospectively. Cases of clinico-histopathological discrepancy underwent blind peer-review, and possible improvements were discussed in the context of the medical literature. A grade for discrepancy was given based on the RADPEER scoring system, and interesting or discrepant cases were examined more closely to identify targets for education. Of the 199 procedures, 4 appendectomies were negative (histologically normal), 182 were positive (primary appendicitis) and 13 were incidental (another primary process caused inflammation). The positive predictive value for pre-appendectomy CT was 91.5 %, and the negative appendectomy rate was 2 %. There were many secondary pathologies, including neoplasia, tuberculosis and endometriosis. Although no CT reports missed a diagnosis that should be made “almost all of the time” and in 96 % of cases, the second, blinded radiologist agreed with the initial assessment, in 3 cases, a missed diagnosis altered clinical management; 2 were “understandable” misses but 1 was not. In five cases, a discrepancy was “understandable” but clinically insignificant. Overall, in comparison to the medical literature, the degree of clinico-histopathological correlation was good. Although identifying areas for improvement was challenging, after a pictorial review of four cases and a discussion of the medical literature, we present our audit results and some valuable learning points for use in the CT assessment of suspected acute appendicitis.

## Introduction

Acute appendicitis is the most common surgical emergency and accounts for one third of patients presenting to the emergency department with an acute abdomen. It has a high morbidity and mortality such that surgeons will tolerate relatively high negative appendectomy rates. The introduction of pre-operative computed tomography (CT) has reduced the number of histologically normal appendectomies [1, 2].

Although acute appendicitis is the most common primary condition of the appendix, other processes can inflame the appendix [3]. These can be primarily inflammatory (e.g. endometriosis, appendiceal diverticulitis, inflammatory bowel disease) or noninflammatory (e.g. neoplasia) in origin. Nonappendiceal conditions such as enteritis, ureterolithiasis, nonappendiceal diverticulitis, mesenteric adenitis, pelvic inflammatory disease and oophoritis can also imitate acute appendicitis.

CT can help discriminate between acute appendicitis and its mimics; however, imaging can still be equivocal such that radiologists may disagree with one another in its interpretation. The RADPEER scoring system (Table 1) uses a peer-review process to grade the level of disagreement between two radiologists [4]. With the intention of enhancing radiologists’ performance, the scoring system helps determine if a discrepancy was avoidable and/or clinically significant and if a missed finding was due to a limitation of the interpreter or the test itself.

**Table 1:** The RADPEER scoring system [4]

Score	Meaning	Optional
1	Concur with interpretation	a. Unlikely to be clinically significant b. Likely to be clinically significant
2	Discrepancy in interpretation/not ordinarily expected to be made (understandable miss)	a. Unlikely to be clinically significant b. Likely to be clinically significant
3	Discrepancy in interpretation/should be made most of the time	a. Unlikely to be clinically significant b. Likely to be clinically significant
4	Discrepancy in interpretation/should be made almost every time—misinterpretation of finding	a. Unlikely to be clinically significant b. Likely to be clinically significant

Alongside CT findings, surgeons must still rely on the clinical picture to decide on the need to operate [5]. Only on receipt of the post-operative histopathology can the diagnosis of acute appendicitis be finally confirmed or refuted.

With a view to improving local practice, this audit was designed to assess the degree of correlation between pre-appendectomy CT and post-appendectomy histology in comparison to the standards set in the literature.

## Material and methods

The audit took place in Changi General Hospital in Singapore between 1 January 2011 and 1 August 2011. Patients included all those that had suspected appendicitis, CT imaging, appendectomy and post-operative histopathological analysis. An Aquilion 64-slice CT scanner (Toshiba Medical Systems, Tokyo, Japan) was used. Images were acquired at 120 kV and 60–100 mAs after a bolus intravenous injection of 70 ml of nonionic (350 mgI/ml) contrast at 1.5 ml/s. Contrast was not used if patients had significant renal impairment or a previous allergic reaction. Where the risks of administering contrast outweighed the benefits, noncontrast scans were performed and the requesting clinicians informed of their limitations. Oral contrast was not used. Axial sections of 3-mm thickness were reconstructed with the FC 18 soft-tissue algorithm. All abdominal and pelvic CT scans included coronal reformats. Saggital reformats were not routinely included but were provided if requested by the attending radiologist.

The CT scans were initially reported by speciality registrars who had completed at least three years of residency training and were FRCR qualified. All reports were checked and signed out by an attending radiology consultant. The senior consultant who performed the blinded RADPEER assessment (AT) is a senior consultant radiologist with over 20 years of experience in gastrointestinal and interventional radiology. Patient demographics, presenting complaints, blood test results, radiological findings and histopathological findings were recorded in an electronic database for analysis.

The defined audit criteria were that, in comparison to the studied literature, the degree of clinico-histopathological correlation between the pre-appendectomy CT and the post-appendectomy histopathology reports should be sufficient and that the number of histologically normal appendectomies should be acceptably low [6]. Our defined audit standards were that the positive predictive value (the proportion of positive CT reports that are histologically confirmed to be primary appendicitis) should be >92 % and that the negative appendectomy rate (the proportion of histologically normal appendectomies) should be <10 % [1, 7]. The data was compared to published standards, and the literature was reviewed. Individual cases were singled out for discussion in the context of the academic literature if there were both interesting and illustrative learning points and RADPEER scores of 2 or more. Areas for improvements were identified for the purposes of educational intervention and improvements in practice.

## Results

One hundred ninety-nine patients met the inclusion criteria, 116 males (58 %) and 83 females (42 %). The average age was 41.7 years (range 14–89 years). All CT reports could not exclude acute appendicitis; 182 positive appendectomies (primary appendicitis) were performed, 106 (58 %) in males and 76 (42 %) in females. Seventeen cases were not primary appendicitis. Four were negative appendectomies (histologically normal), representing a positive predictive value for pre-appendectomy CT of 91.5 % and a negative appendectomy rate of 2 %, respectively. The remaining 13 (6.5 %) were incidental appendectomies (appendiceal inflammation secondary to another primary process); 8 were appendiceal or periappendiceal inflammation mimicking acute primary appendicitis, and 5 were appendiceal inflammation secondary to a primarily noninflammatory process (Table 2). The impact of intravenous contrast on the rates of negative or incidental appendectomies could not be established as all 17 had contrast-enhanced CT scans.

**Table 2:** Frequency of conditions mimicking acute appendicitis

Condition mimicking appendicitis	Number
Primarily inflammatory	
Appendiceal diverticulitis	4
Early pelvic inflammatory disease	1
Endometriosis	1
Enteritis	1
Gastrointestinal tuberculosis	1
Primarily noninflammatory	
Adenocarcinoma	2
Carcinoid tumour	2
Mucinous cystadenoma	1

No CT reports missed a diagnosis that should be made almost all of the time (RADPEER score “4”). In 96 % of cases (191), the second, blinded radiologist agreed with initial reporter’s assessment (RADPEER score “1”). There were three cases in which a missed diagnosis altered the clinical management of the patient (cases 2–4). Two were “understandable misses” (RADPEER score “2b”; cases 3 and 4) but 1 was not (RADPEER score “3b”; case 2). There were five cases (3 %) where a diagnosis was missed but it was an understandable miss and not clinically significant (RADPEER score “2a”; case 1).

## Discussion

Acute appendicitis is a common and potentially hazardous condition. Although waiting for a diagnostic CT can cause a fatal delay in undergoing appendectomy, it has been shown to be an effective diagnostic tool [1]. The implementation of pre-operative CT imaging has reduced the negative appendectomy rate from around 10–20 % to 5 % [7, 8]. In this audit, the negative appendectomy rate was 2 %; one of the audit standards was achieved. The positive predictive value of pre-appendectomy CT was 91.5 %, falling just short of our defined audit standard. In 13 cases, an alternative diagnosis was discovered incidentally, post-operatively. These were mainly rare appendiceal conditions that can mimic acute appendicitis clinically and radiologically (Table 2). In most of them, the second, blinded radiology report concurred with the first such that the discrepancy was an objective imperfection of computed tomography and not the interpreting radiologist (RADPEER score 1). In only 3 of 199 appendectomies (1.5 %) was there a misinterpretation of the CT findings that was felt to have altered clinical management (cases 2–4). Radiologists must be aware of the full spectrum of appendicitis mimics and their associated CT findings. Four cases were singled out for more detailed discussion (cases 1–4). Reviewing cases 1–4 in the context of the published literature, they tend to present with atypical features in either the history, examination, blood tests or imaging results. It is, therefore, vital that radiologists are meticulous in their search for possible secondary pathologies, especially if unusual features are present. This may offer a better alternative diagnosis and prevent error. For example, in the discussed cases, if the suggestion of neoplasia, enteritis or gynaecological pathology had been raised on imaging, there may have been an alternative management plan more appropriate than urgent appendectomy (cases 1–4).

The learning points identified in this audit were presented at the annual departmental audit meeting and at both a Singaporean and British national radiology conference. Other interventions included self-directed and consultant-led education, modification of the current appendicitis CT protocol to include more senior guidance and the addition of an ongoing RADPEER peer-review scoring system to identify further areas for improvement.

There are three important limitations to this study. Firstly, in excluding patients with suspected appendicitis who underwent CT without appendectomy, we cannot calculate the sensitivity, specificity and negative predictive value of CT in suspected acute appendicitis. These particular statistics can, therefore, not be compared to the published literature, and we are limited to the positive predictive value and negative appendectomy rate. To overcome this would require a prospective rather than retrospective study, following up patients with suspected appendicitis who undergo CT but not appendectomy. Secondly, we also excluded patients who underwent appendectomy without pre-operative CT. This skews our data set towards ambiguous or equivocal cases, as patients who go to theatre without prior imaging would presumably have a more classical clinical picture. A final limitation is in the use of positive predictive value and negative appendectomy rate in assessing the quality of radiology reporting. These measures are suboptimal as they are compounded by two other factors: firstly, by how they are defined and secondly, by individuals other than the interpreting radiologists. Defining a positive appendectomy as an appendectomy for primary appendicitis ignores the fact that appendectomy is an appropriate management plan for conditions other than primary appendicitis; the positive predictive value does not account for these and will, therefore, tend to be lower than expected. This may account for why our second standard was not achieved. Secondly, the positive predictive value is affected by not only the radiologist but also the referring clinician, the surgeon, the radiographer and the histopathologist. Improving the positive predictive value and negative appendectomy rates is, therefore, not solely the responsibility of the interpreting radiologists, but other members of the multidisciplinary team as well. To overcome this, rather than using the positive predictive value and negative appendectomy rate, we would instead compare our RADPEER scores to the standard set by the literature (e.g. a large, multicentre sample of RADPEER scores). This would eliminate these confounding factors and also take into account the imperfections of the test itself, the intrinsic limitations of computed tomography in suspected acute appendicitis. However, no such sample exists. Therefore, the next best standard is the positive predictive value and negative appendectomy rate. Any further interventions should also target other relevant members of the multidisciplinary team.

## Conclusion

Compared to the consensus of peer-reviewed publications, the correlation between the pre-appendectomy CT and post-appendectomy histology was good. Identifying areas for improvement was possible through detailed assessment of four case histories alongside the published literature. These provide some important illustrative learning points. Particular attention is paid to the other pathologies that can mimic acute appendicitis, in which the recognition of certain CT findings could trigger important changes in patients’ management plans.

## Cases 1

**Fig. 1:**
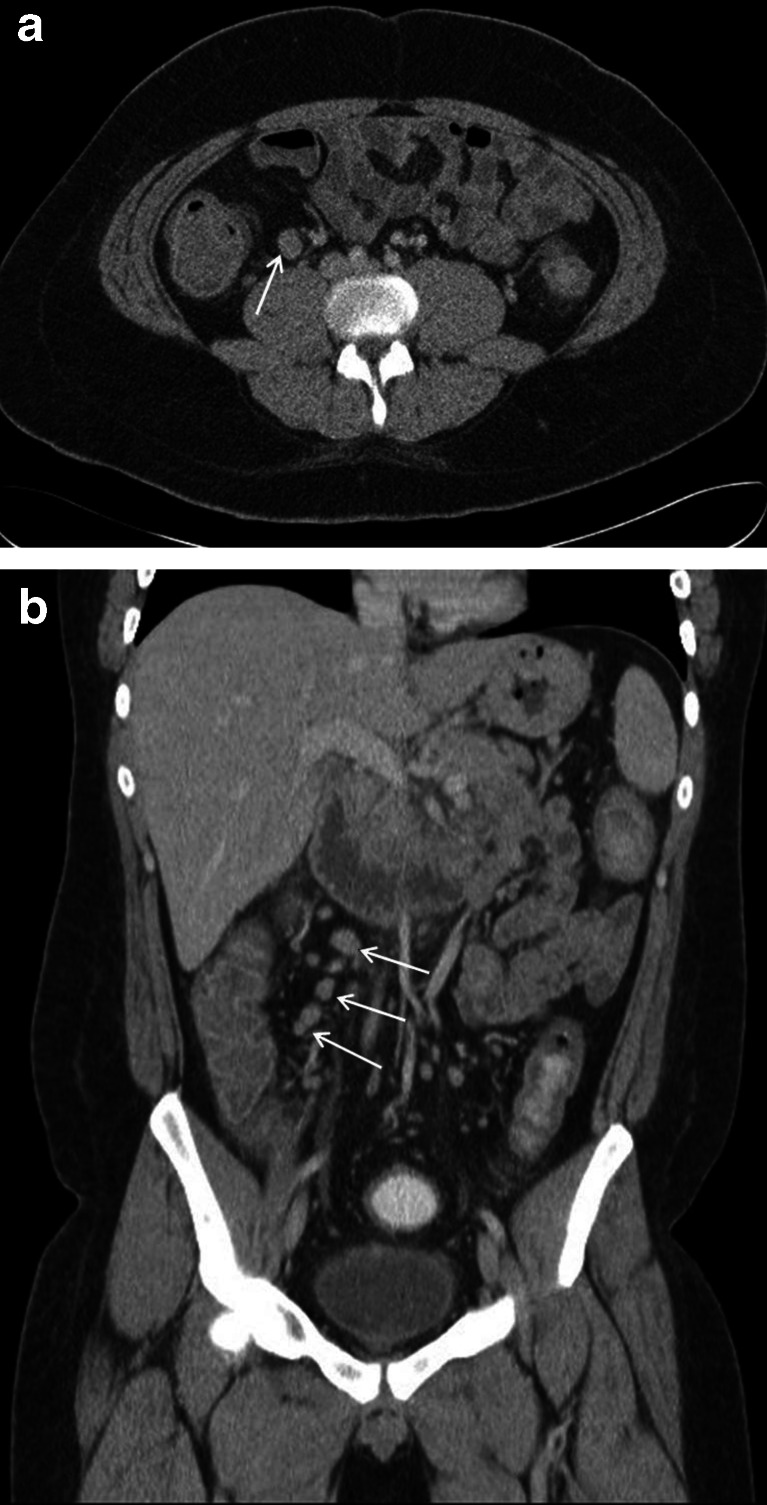
A 16-year-old male with mesenteric lymphadenitis mimicking acute appendicitis (case 1). Contrast-enhanced axial (**a**) and coronal (**b**) images demonstrate prominent enlarged ileocolic lymph notes (*white arrows*). The appendix could not be identified due to paucity of intra-abdominal fat.

A 16-year-old male presented to the emergency department with a 3-day history of crampy epigastric and right iliac fossa pain. There was no fever, rigors or rebound tenderness. The white cell count was 13.9 × 10^9^/L. Due to paucity of abdominal fat, the appendix could not be identified on CT; however, there were enlarged right ileocolic lymph nodes and free pelvic fluid (Fig. 1a, b). “Appendicitis could not be excluded”, and the patient underwent appendectomy, but the appendix was histologically normal. The ultimate diagnosis was mesenteric lymphadenitis; however, this was felt to be an “understandable miss” that was, given the strong clinical picture, unlikely to have affected management. It was, therefore, allocated a RADPEER score of 2a. Nikolaidis et al. found that “of [their] 46 patients with a nonvisualized appendix, only 1 (2 %) was found to have acute appendicitis” and that “nonvisualisation of the appendix, even when there is a paucity of abdominal fat, may safely exclude acute appendicitis, as long as no secondary CT findings of appendicitis are present” [9]. Two further studies concur; however, the negative predictive value of appendiceal nonvisualisation regardless of other appendicitis-related changes is yet to be assessed [10, 11].

## Cases 2

**Fig. 2:**
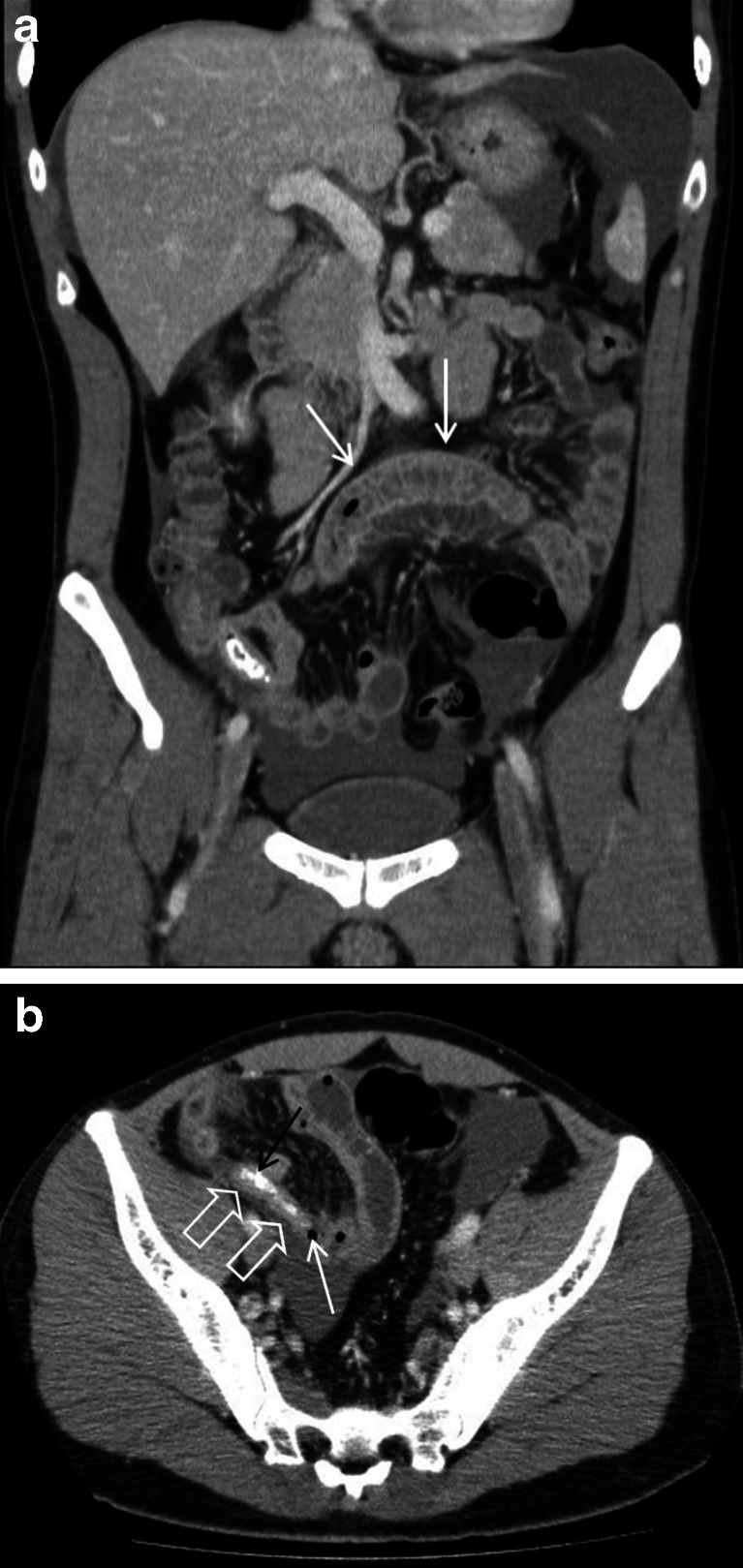
**a** A 32-year-old male with enteritis masquerading as acute appendicitis (case 2; see **b**). Contrast-enhanced coronal images demonstrate a fluid-filled small bowel loop with mural thickening and enhancement in keeping with enteritis (*white arrows*). Ascites is noted within the perisplenic region and pelvis. **b** A 32-year-old male with enteritis (case 2; see **b**). Contrast-enhanced axial CT image demonstrates a small air locule (*white arrow*) at the tip of a blind-ending tubular structure (*open white arrows*) within the right iliac fossa with a slightly thickened and enhancing wall, suggesting possible perforation of an acutely inflamed appendix. Intraluminal dense material was in keeping with an appendicolith (*black arrow*)

A 32-year-old male presented with a 1-day history of crampy abdominal pain that started on the left but migrated suprapubically and to the right iliac fossa. There was rebound tenderness and guarding, and the white cell count was 12 × 10^9^/L. On CT, there was “fluid in the pelvis, right iliac fossa (RIF), perihepatic and splenic regions, peritoneal enhancement in the RIF and right pelvic sidewall, a small air locule adjacent to an inflamed appendix and an appendicolith in the appendix” (Fig. 2a, b). There were also “dilated, prominent, fluid-filled small bowel loops” that were thought to represent localised ileus. The diagnosis of acute appendicitis was suggested, and perforation could not be excluded; however, after appendectomy, the histological findings demonstrated “no ulceration, transmural inflammation or serosal exudate to indicate acute appendicitis”. The second, blinded radiologist felt that the disproportionate volume of free fluid and the presence of inflamed small bowel should have suggested another pathology (e.g. enteritis) and that this was overlooked initially. This was likely to be clinically significant as an ascitic tap may have followed instead, followed by primarily conservative or medical rather than surgical management. It was, therefore, allocated a RADPEER score of 3b. The reliability of the appendicolith especially on CT is often overemphasised. Appendicoliths are present in only 23–46 % of those with acute appendicitis; the presence of an appendicolith has no diagnostic significance, and it may be an incidental finding in asymptomatic patients. No research has suggested that those with an appendicolith are at increased risk of appendicitis [12–14].

## Case 3

**Fig. 3:**
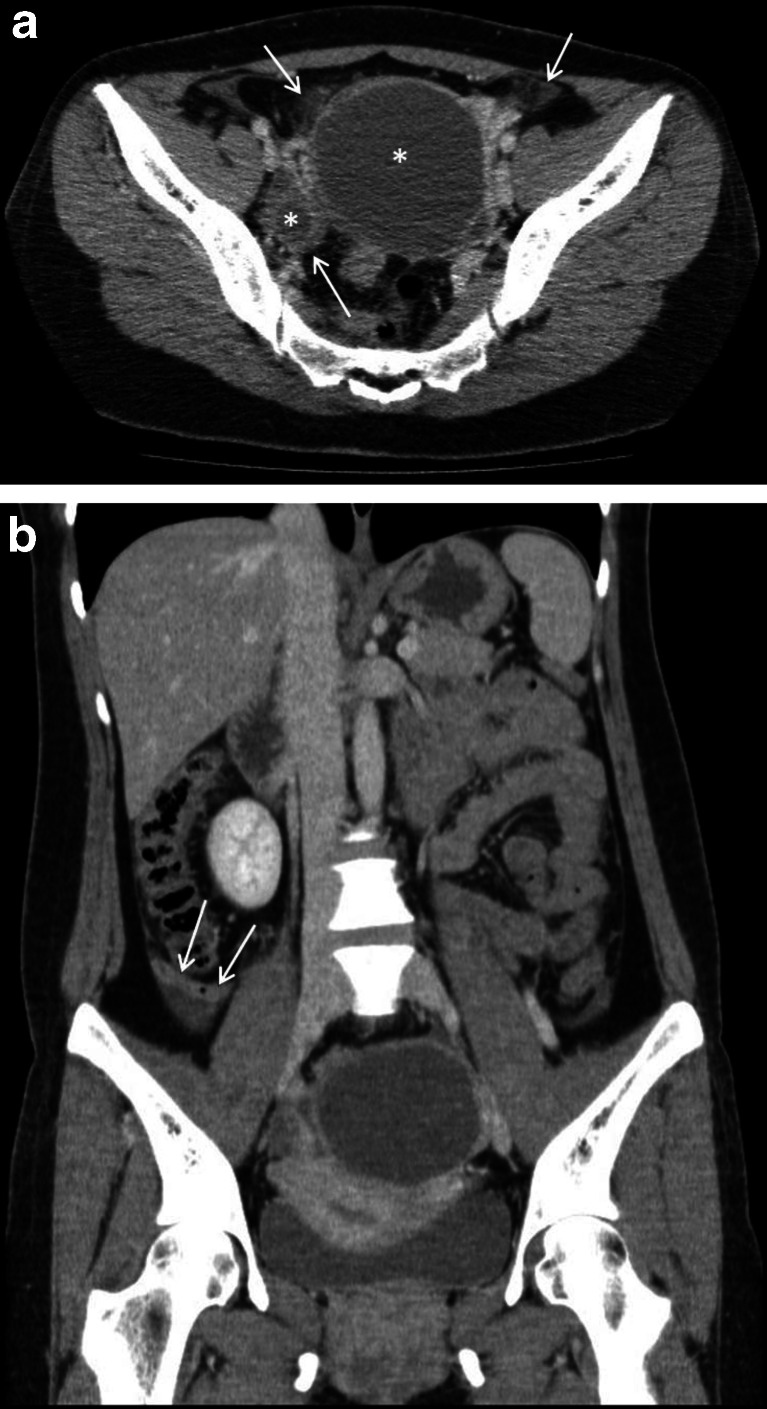
**a** A 35-year-old female with oophoritis in early pelvic inflammatory disease mimicking an acute appendicitis (case 3; see **a**). The contrast-enhanced axial CT scan image reveals incidental bilateral ovarian cysts, larger on the left (*white asterisks*). There are small pockets of free fluid and inflammatory fat stranding surrounding the ovarian cysts (*white arrows*). **b** A 35-year-old female with oophoritis in early pelvic inflammatory disease (case 3; see **a**). In this contrast-enhanced coronal CT image, there is a suggestion of mild focal mural thickening and enhancement of the retrocaecal appendix (*white arrows*), associated with a small amount of periappendiceal fluid

A 35-year-old female presented with a 1-day history of nausea, vomiting, diarrhoea and left iliac fossa pain that migrated to the right side. The white cell count was 15 × 10^9^/L, and a urine dipstick was positive for microscopic haematuria and leucocytes. The CT reported “focal mural thickening, enhancement of the appendix just proximal to the tip and free fluid around the appendix, in the paracolic gutters and within the pouch of Douglas” suggesting that “in the appropriate clinical context, early appendicitis could not be excluded” (Fig. 3a, b). The report added that “bilateral ovarian cysts seen on CT warrant follow-up pelvic ultrasound”. Post-operative histopathology reported mild mucosal congestion and ulceration but no acute appendiceal inflammation. The second blinded radiologist noted bilateral inflammation around the ovarian cysts such that the pathology was more likely to be primarily gynaecological rather than appendiceal (e.g. ovarian pathology, pelvic inflammatory disease). Although this was felt to be an understandable miss, such a finding would have necessitated an alternative management plan, and therefore, this was allocated a RADPEER score of 2b. As for the previous case, this case highlights the importance of considering an alternative diagnosis, especially if the history, examination and/or investigation findings are unusual or equivocal.

## Case 4

**Fig. 4:**
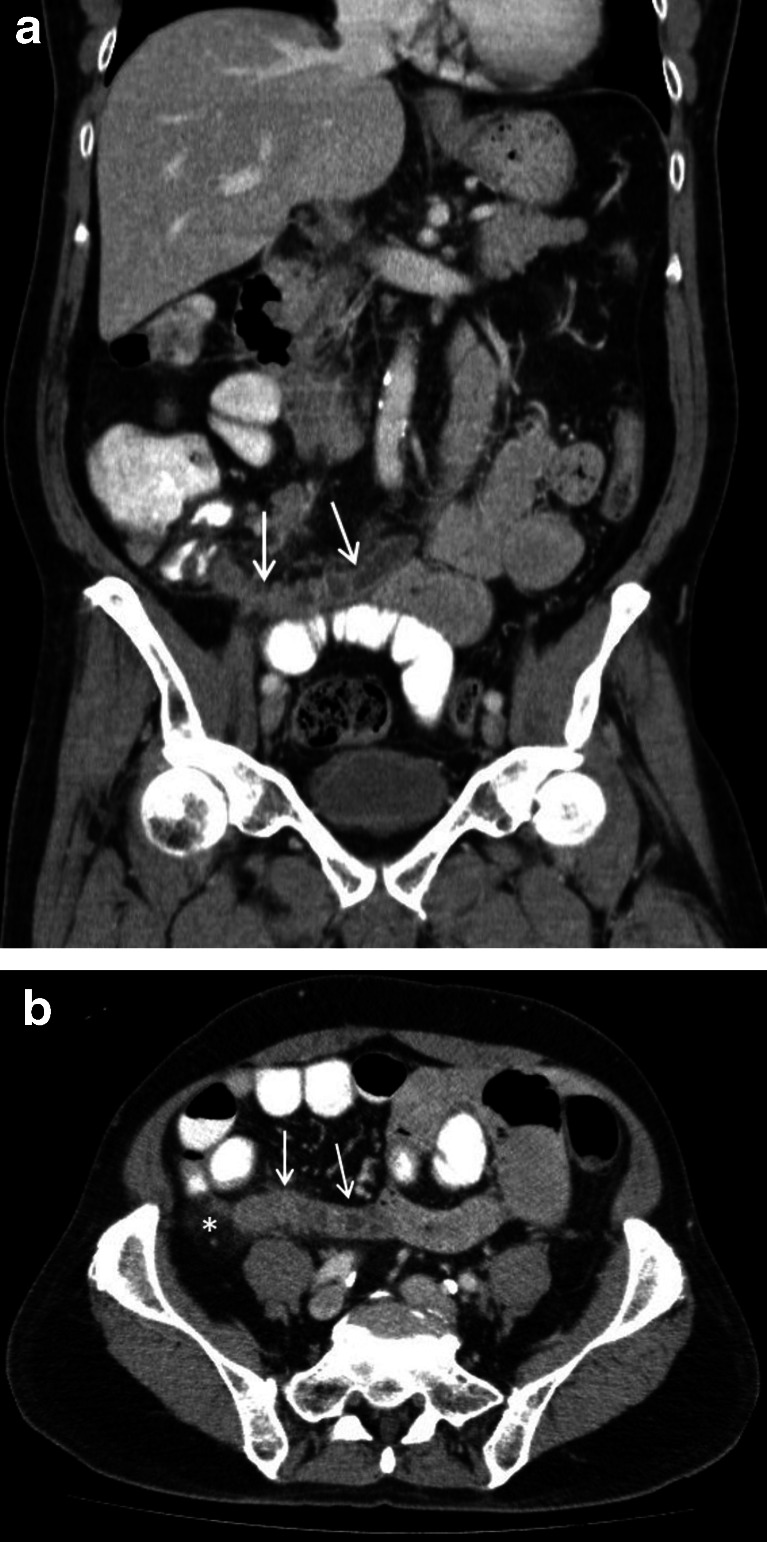
A 64-year-old male with moderately differentiated adenocarcinoma of the appendix (case 4). The coronal (**a**) and axial (**b**) contrast-enhanced CT scan images reveal a dilated, fluid-filled appendix with mural thickening and enhancement (*white arrows*). There is also evidence of periappendiceal inflammatory fat stranding (*white asterisk*) which is best appreciated in the axial image

A 64-year-old male presented with a 1-day history of continuous, crampy, bilateral, lower abdominal pain. The radiological impression was in keeping with acute appendicitis, with a “large (20 mm), distended, fluid-filled appendix, periappendiceal fat stranding, ileocolic lymph node enlargement and dilated small bowel loops in keeping with ileus”. An “irregular appendix wall showing heterogenous enhancement” was felt to be gangrenous (Fig. 4a, b). The post-operative histopathology report reported a moderately differentiated adenocarcinoma with lymphovascular invasion in association with several sessile adenomatous polyps and acute secondary appendicitis. As the appendiceal resection margins were involved, a right hemicolectomy and lymph node clearance were performed 2 weeks later. The second, blinded radiological evaluation concluded that this was an understandable miss that affected clinical management and was, therefore, allocated a RADPEER score of 2b. Studies have reported that up to 1 % of appendectomies are neoplastic, of which 18–50 % are malignant. Such patients present in their 5th and 6th decades, usually with acute appendicitis. When diagnosed intraoperatively, a search should be conducted for sites of metastatic disease. Appendectomy is sufficient in most cases; however, in this case, it was not and required a reoperation [15]. Although it is difficult to make a definitive pre-operative diagnosis, “morphological changes, such as cystic dilatation or a focal soft-tissue mass are present in the majority of cases, and an appendiceal diameter of >15 mm should be viewed with suspicion” [16, 17].
